# Optimization and validation of a highly sensitive method for determining glyphosate in human urine by solid-phase extraction and liquid chromatography with tandem mass spectrometry: a methodological study

**DOI:** 10.1186/s12199-020-00918-w

**Published:** 2020-12-12

**Authors:** Hiroshi Nomura, Risa Hamada, Isao Saito, Kunihiko Nakane, Ritsuko Sawa, Miwa Ukai, Eiji Shibata, Mitsuo Sato, Michihiro Kamijima, Jun Ueyama

**Affiliations:** 1 Department of Biomolecular Sciences, Field of Omics Health Sciences, Nagoya University Graduate School of Medicine, 1-1-20 Daiko-minami, Higashi-ku, Nagoya, Aichi 461-8673 Japan; 2 Okazaki City Public Health Center, 2-1-1 Wakamiya-cho, Okazaki, Aichi 444-8545 Japan; 3 The Association for Preventive Medicine of Japan, 1-19-10 Mouri, Koto-ku, Tokyo, 135-0001 Japan; 4 Aichi Medical University Health Service, 1-1 Yazakokarimata, Nagakute, Aichi 480-1195 Japan; 5 Department of Host Defense Sciences, Field of Omics Health Sciences, Nagoya University Graduate School of Medicine, 1-1-20 Daiko-minami, Higashi-ku, Nagoya, Aichi 461-8673 Japan; 6 Department of Occupational and Environmental Health, Nagoya City University Graduate School of Medical Sciences, 1 Kawasumi, Mizuho-cho, Mizuho-ku, Nagoya, Aichi 467-8601 Japan

**Keywords:** Biomonitoring, Glyphosate, Human urine, LC-MS/MS, Solid-phase extraction

## Abstract

**Background:**

Glyphosate and its salt formulations are nonselective herbicides that have been extensively used worldwide, both for residential and agricultural purposes. The possible carcinogenicity and teratogenicity of glyphosate remain to be elucidated. We developed a sensitive and high-throughput analytical method for urinary glyphosate using liquid chromatography-tandem mass spectrometry with the aim of contributing to glyphosate exposure assessment in epidemiological studies.

**Methods:**

After urine dilution (creatinine matching dilution to 0.05 g creatinine/L), glyphosate was extracted using two types of solid phase extraction columns (SCX and NH2) with automated sample preparation instruments. The eluate was dried and dissolved in the mobile phase, followed by liquid chromatography-tandem mass spectrometry analysis. The optimized method was applied to urine samples obtained from 54 Japanese adults and children.

**Results:**

The results from the validation study demonstrated good recoveries (91.0-99.6%), within- and between-run precisions (< 15%), low detection limits (0.1 μg/L), and lower limit of quantification (0.3 μg/L). The detection frequency and median concentration of the urinary glyphosate in Japanese subjects were 59% and 0.25 μg/L (0.34 μg/g creatinine).

**Conclusions:**

Our reliable determination method was successful in measuring urinary glyphosate concentration. Moreover, this is the first biomonitoring report of urinary glyphosate levels in the Japanese general population.

## Introduction

Glyphosate and its salt formulations are nonselective herbicides that have been extensively used worldwide, both for residential and agricultural purposes [1]. Not only in countries where their use is allowed for cultivation of genetically modified glyphosate-resistant crops such as wheat and corn but also in Japan and other counties, glyphosate is frequently used in agricultural fields, playgrounds, parking areas, and roads for weed control [2]. In Japan, the total domestic shipment of glyphosate, which is mainly composed of glyphosate isopropylamine salt and glyphosate potassium salt, has been growing steadily from 2300 tons in 2000 to 5670 tons in 2017 [3].

Whether the widely used glyphosate poses a possible risk to human health is a controversial matter. In 2015, glyphosate was classified as 2A “possibly carcinogenic to humans” by the International Agency for Research on Cancer [4]. On the other hand, several regulatory agencies [5–7] reviewed the scientific data and denied the carcinogenicity of glyphosate. More recently, a large-scale cohort study focused on the relationship between glyphosate exposure and health risks. The Agricultural Health Study, which is a prospective cohort study in North Carolina and Iowa, reported that a relationship was evident between glyphosate exposure and the risk of acute myeloid leukemia [8]. In view of the prospective, large-scale increase in the glyphosate usage level worldwide, nationwide studies covering exposure assessment of glyphosate followed by risk assessment are needed in Japan as well.

Generally, exposure assessment approaches are as follows: questionnaire/historical information, environmental monitoring (air, water, food, and soil), and/or human biomonitoring (HBM). Human biomonitoring is a unique approach for assessing the internal dose of chemicals not only in occupational but also in environmental settings, usually based on the analysis of human specimens (e.g., urine or blood). It plays a key role in providing quantitative information of individual exposure levels to epidemiological studies. In the case of glyphosate exposure estimation, HBM of urinary glyphosate has been used practically, because approximately 20-30% of glyphosate dose is excreted in urine within 48 h after the exposure [7]. In 2017, the German Federal Institute for Risk Assessment (BfR) conducted a risk assessment of glyphosate, using the HBM of glyphosate in urine obtained from over 2000 samples [9]. Moreover, some glyphosate HBM data from Germany [10], Denmark [11], Sri Lanka [12], the USA [13], and Ireland [14] (less than 100 urine samples) have also been reported, which is partly promoted by the development of glyphosate determination using urine.

In recent years, urinary glyphosate levels in the general population have been reported using various analytical methods (Table 1). Ligand binding assays, such as the enzyme-linked immunosorbent assay (ELISA), have some merits in terms of simple, rapid, and high-throughput data measurement in 96-well plates [19]. Chromatographic assays using gas chromatography (GC)-mass spectrometry (MS/MS) [10] and high-performance liquid chromatography (LC)-MS/MS have also been reported [14]. Each determination method has obvious benefits and drawbacks in terms of simplicity, reliability, and sensitivity. Given that large epidemiological studies and national surveys have been undertaken to reveal the chemical exposure risk to human health, there is an urgent need to further develop a high-throughput and sensitive method for the quantification of urinary glyphosate from general populations. Moreover, additional information is needed to reveal the characterization of urinary glyphosate concentration as exposure biomarkers.

**Table 1:** Analytical methods for detecting glyphosate in human urine samples

References	Sample preparation	Instrument for separation analysis	Limit of detection (μg/L)
Acquavella et al. [15]	Solid-phase extraction, derivatization	HPLC	1
Biagini et al. [16]	Derivatization	FCMIA	0.9
Curwin et al. [17]	Derivatization	FCMIA	0.9
Jayasumana et al. [12]		ELISA	0.6
Jensen et al. [18]	Dilution of urine	LC-MS/MS (ESI)	0.023
Connolly et al. [14]	Solid-phase extraction	LC-MS/MS (ESI)	0.5
Conrad et al. [10]	Derivatization	GC-MS/MS (NCI)	0.1 (LLOQ)

*HPLC* high-performance liquid chromatography, *FCMIA* fluorescence covalent microbead immunosorbent assay, *ELISA* enzyme-linked immunosorbent assay, *LC* liquid chromatography, *GC* gas chromatography, *MS* mass spectrometry, *ESI* electrospray ionization, *NCI* negative chemical ionization, *LLOQ* lower limit of quantification

Currently, there are limited HBM data available for glyphosate exposure assessment in Japan. This study aimed to develop a fully validated quantitative method for urinary glyphosate, which is applicable to the general population with no documented exposure to glyphosate using liquid chromatography-tandem mass spectrometry (LC-MS/MS).

## Methods

### Chemicals and reagents

A standard reagent of glyphosate (99.3% purity) was purchased from FUJIFILM Wako Pure Chemical Co. (Osaka, Japan) and glyphosate-^13^C_2_. ^15^N was obtained from Toronto Research Chemicals Inc. (Toronto, Canada) and used as an internal standard (IS). Ultrapure water (LC-MS grade), methanol (LC-MS grade), formic acid, acetic acid, 1 mol/L ammonium formate solution, and 6 mol/L hydrochloric acid were purchased from FUJIFILM Wako Pure Chemical Co. (Osaka, Japan). InfinityLab deactivator additive (composed of 5 mmol/L of medronic acid, MA) was obtained from Agilent Technologies, Inc. (CA, USA). Ammonia solution (28% in water) was obtained from Tokyo Chemical Industry Co., Ltd. (Tokyo, Japan). Water was distilled and deionized to 18 MΩ with a Milli-Q system (Millipore, MA, USA). A polymeric sulfonic acid bonded silica solid-phase extraction (SPE) product, ISOLUTE®-96 SCX 25 mg fixed well plate (Biotage, Uppsala, Sweden) and the polymeric aminopropyl bonded silica SPE product, ISOLUTE®-96 NH2 100 mg fixed well plate (Biotage, Uppsala, Sweden), was used for glyphosate extraction from urine samples. The SPE was performed using an automated system with plate formats called Extrahera™ (Biotage, Uppsala, Sweden).

### Pooled urine samples and standard solutions

Pooled urine was collected from 96 healthy volunteers (63 males and 33 females), and was used for all optimization studies, matrix-matched calibration curves, and validation assays. Standard glyphosate and glyphosate-^13^C_2_, ^15^N were dissolved to a concentration of 1000 mg/L in water and diluted with water to prepare working reference solutions at designated concentrations. The glyphosate solution was stored at 4 °C, and the glyphosate-^13^C_2_, ^15^N solution was stored at −40 °C in the dark, which was used without a freeze-thaw cycle.

### Sample preparation procedure

**Fig. 1:**
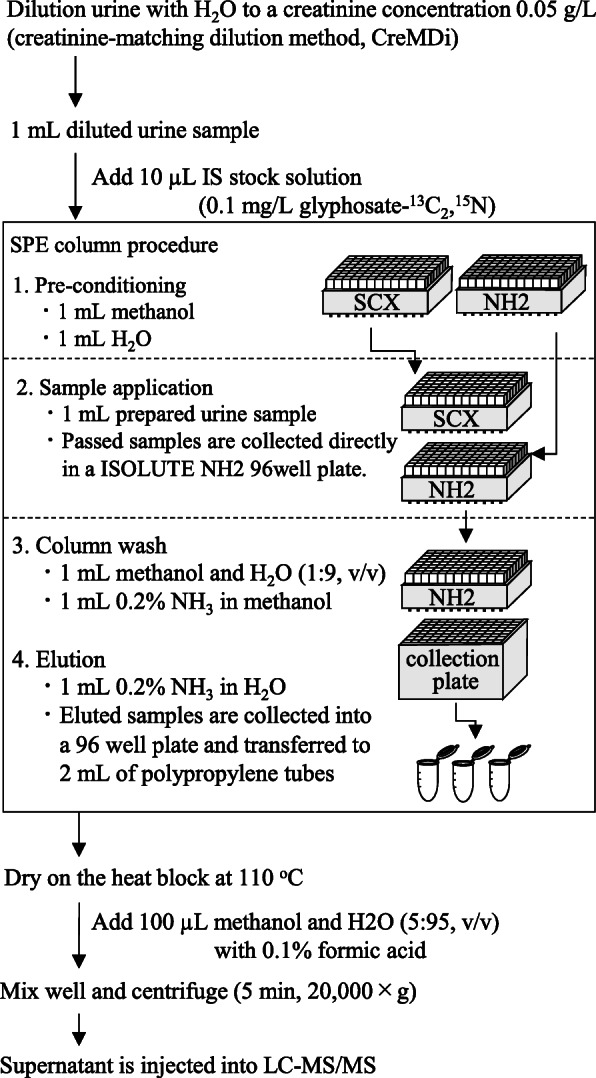
Established analytical method for urinary glyphosate

A flow chart of the sample preparation procedure for the measurement of urinary glyphosate is shown in Fig. 1. Initially, all urine samples were diluted to a creatinine concentration of 0.05 g/L (creatinine-matching dilution to 0.05 g/L, abbreviated to CreMDi_0.05_). This dilution was performed automatically using an OT-2 pipetting robot (NY, USA). One milliliter of the diluted urine samples was pipetted into a 96-well plate (each well volume was 2 mL), and 10 μL of IS solution (0.1 mg/L glyphosate-^13^C_2_, ^15^N) was added. After gentle shaking, the urine sample was subjected to SPE and was performed using the ExtraheraTM automation system (Biotage, Uppsala, Sweden) equipped with a nitrogen positive pressure unit.

Preconditioning of the SCX cartridge and the NH2 cartridge was achieved by washing with 1 mL of methanol, followed by conditioning with 1 mL of H_2_O. One milliliter of the prepared urine sample was loaded into the conditioned SCX cartridge. The passed through sample was directly collected in an NH2 cartridge. Moreover, the solutions were passed through the NH2 cartridge, and the target compounds were absorbed into the column. The NH2 cartridge was washed with 1 mL of a solution of methanol and H_2_O (1:9, v/v), followed by washing with 1 mL of a solution of methanol and 28% NH_3_ aqueous solution (140:1, v/v). Then, the target compounds were eluted with 1 mL of a solution of H_2_O and 28% NH_3_ aqueous solution (140:1, v/v; namely 0.2% NH_3_ solution).

The eluted fraction was transferred to polypropylene tubes and dried on a heat block at 110 °C. The residuals were dissolved in 100 μL of a solution of methanol and H_2_O (5:95, v/v) with 0.1% formic acid with 0.1% deactivator additive (5 μmol/L MA), and centrifuged at 20,000×*g* for 5 min after gentle vortexing. Finally, the supernatant was analyzed with LC-MS/MS.

### Chromatography and mass spectrometry

The LC-MS/MS analysis was run on an Agilent 1260 Infinity Binary LC system coupled with an Agilent 6430 triple quadrupole mass spectrometer (Agilent Technologies, Inc., CA, USA). The LC operating conditions were as follows: LC column, Scherzo SM-C18 MF (Imtakt, Kyoto, Japan), 100 × 2 mm i.d., 3 μm silica; mobile phase A, methanol and H_2_O (5:95, v/v) containing 0.1% of formic acid with 0.1% of the deactivator additive (5 μmol/L MA), mobile phase B; methanol and 20 mmol/L ammonium formate solution (20:80, v/v) with 0.1% of the deactivator additive (5 μmol/L MA); gradient condition of mobile phase B, 0% (0-5 min)-90% (5-10 min)-0% (10-12 min); the total flow rate, 0.2 mL min^−1^; total run time per sample, 12 min; and injection volume, 10 μL. Mobile phase A (40 μL) was injected 3 times (run time was 0 min) between each sample analysis to prevent carry-over contamination.

The MS/MS was operated with an electrospray ionization (ESI) source in positive ion mode with multiple reaction monitoring (MRM). The nebulizer gas pressure, source temperature, and gas flow were 50 psi, 350 °C, and 10 L/min, respectively. The capillary voltage was 4000 V (positive mode), and high-purity nitrogen gas was used in the collision cell. Table 2 shows the optimized MRM parameters, and retention times for glyphosate and IS. The chromatograph and mass spectrogram data were collected using the Mass Hunter Software Workstation (Agilent Technologies).

**Table 2:** Compound-specific mass spectrometer settings

Compounds	Fragmentor (V)	Collision energy (eV)	Precursor ion (*m/z*)	Product ion (*m/z*)	Retention time (min)
Glyphosate	70	6	170	88.1 (Q)	3.4
	70	20	170	60.1 (C)	3.4
	70	28	170	42.2 (C)	3.4
Glyphosate-^13^C_2_, ^15^N	70	7	173	91.1 (C)	3.4
	70	7	173	62.2 (Q)	3.4

*Q* quantification ion, *C* confirmation ion

### Assay validation

The present bioanalytical method was validated in terms of matrix effects, precision, extraction recovery, linearity, limit of detection (LOD), lower limit of quantification (LLOQ), prepared sample stability, freeze-thaw stability, long-term stability, and robustness.

The matrix effects are represented as the absolute matrix factor and relative matrix factor. The absolute matrix factor was calculated by dividing the peak area of glyphosate in the urine matrix by the peak area of glyphosate standard solution dissolved in the LC mobile phase A. The relative matrix factor was represented by dividing the glyphosate/IS peak area ratio in the matrix by those of glyphosate standard solution in the mobile phase A. Glyphosate standard solution and IS solutions were spiked just before the LC-MS/MS analysis (final concentrations were 1 μg/L of urine). The matrix factors were calculated using 10 individual urine samples (8 male and 2 female adults) to evaluate the range of matrix effects. Tenfold dilution urine from the 10 individuals was also used for the matrix factor calculation to compare the creatinine-matching dilution method. The average creatinine concentration after tenfold dilution was 0.08 g/L.

The within-run precision was examined by determination of pooled urine spiked with glyphosate at concentrations of 0.33, 0.67, and 1.33 μg/L (*n* = 6). The between-run precision was evaluated at concentrations of 0.33, 0.67, and 1.33 μg/L (*n* = 2) for five consecutive days.

Absolute recovery rates were estimated at three concentration levels: 0.33, 0.67, and 1.33 μg/L (*n* = 3). Recovery rates were calculated by comparing the peak areas derived from the following two sets of procedures. Samples in the first set were spiked with glyphosate prior to sample preparation. The second set of samples was spiked just before LC-MS/MS analysis.

Calibration curves using urine were represented by glyphosate/IS. The peak area ratio versus the concentrations of glyphosate ranged from 0.17 to 2 μg/L (0.17, 0.33, 0.5, 1, 2, and 5 μg/L). The concentration ranges were designed referring to urinary glyphosate concentrations in previous reports [10, 14]. The linearity of the calibration curve was determined by linear regression analysis. Calibration curves with coefficients of determination (*r*^*2*^) ≥ of 0.97 were considered linear.

The LOD and LLOQ were derived from the assumption of signal-to-noise ratios of 3 and 10, respectively. In addition, the LLOQ of within-run precision was defined as less than 20% (relative standard deviation, %RSD).

The robustness of the developed method was tested by continuous analysis of the prepared samples. We used glyphosate-spiked urine at a concentration of 0.67 μg/L, and after the sample preparation procedure, the samples were analyzed eighty-six times in a row. We evaluated the %RSD of glyphosate/IS peak area ratios for all analyses.

### Sample stability

The stability of the prepared samples was tested through a duplicate assay at a concentration of 0.67 μg/L glyphosate. The prepared sample was stored in an LC autosampler at 4 °C and analyzed at five-time points as follows: 0 (immediately), 18, 24, 48, and 72 h. Stability was assessed by comparing the glyphosate/IS peak area ratios of samples stored for 18, 24, 48, and 72 h with those of samples stored for 0 h.

Freeze-thaw stability of glyphosate in urine was evaluated at concentrations of 0.67 μg/L. Each freeze-thaw cycle consisted of a minimum of 12 h freezing at −80 °C followed by a complete thaw in tap water running at room temperature for 10 min. Samples were analyzed after the third freeze-thaw cycle. The stability was evaluated by comparing glyphosate/IS peak area ratios with those of samples that did not undergo freeze-thaw cycles (*n* = 3).

The long-term stability of glyphosate in urine was estimated using urine samples spiked at a concentration of 0.67 μg/L. We stored the urine samples for 1 week at ambient temperature (25 °C), and those for 1 week and 1 month at 4 °C and −80 °C, and analyzed their glyphosate concentrations. The peak area ratios of the stored samples were compared with those of non-stored and spiked urine samples (*n* = 3).

### Application of methods to urine samples

Our method was applied to human spot urine samples obtained from children (group A), adults (group B), and farmers (group C). Prior to enrollment in the study, an informed consent form was signed by each subject or the parent giving the right to the use of urine samples for research purposes. Morning voids of children were collected from 3-year-old children (10 males and 10 females) who attended a municipal health check program in a suburban area of Aichi, the central region of Japan. Group B consisted of 24 adults composed of 14 males and 10 females (age range 31-63 years) living in the Kyushu region of Japan. The adult samples (group C) were composed of eight male and two female farmers (age range 44-73 years) living in the Kyushu region. Collected spot urine samples were transported at 4 °C, and then stored at −80 °C until analysis.

Creatinine analysis was completed on all samples using a previously reported method [20] with a high-performance liquid chromatograph equipped with a UV detector. Undetectable urinary concentrations of glyphosate were estimated as the LOD value divided by the square root of 2 [21] to calculate geometric means (GM).

## Results

### Optimization of the LC column and mobile phase

**Fig. 2:**
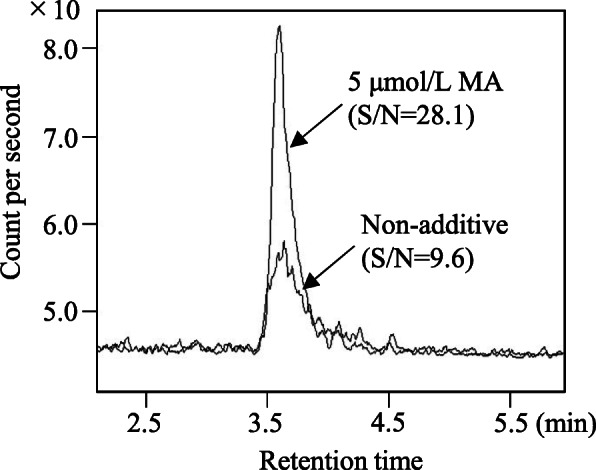
Chromatograms peaks of 1 μg/L glyphosate standard solution resulting from mobile phase with or without 5 μmol/L of medronic acid (MA)

Given that glyphosate is an ionic and highly water-soluble compound, it is difficult to hold it using a common LC separation column such as a reversed-phase column. Therefore, we selected the ODS column (Scherzo SM-C18 MF) consisting of C18, weak anion, and weak cation ligands as the LC column. In addition, as glyphosate is a metal coordination compound with a phosphate group in its structure, it tends to show poor peak shape and sensitivity by interacting with the metal of the steel column, metallic pipes, and the solution of the mobile phase. With the aim of minimizing these effects and improving the detection limit of glyphosate, we used MA deactivator additive as the LC mobile phase additive. The use of MA improved glyphosate peak intensity (Fig. 2).

### Optimization of sample preparation

**Fig. 3:**
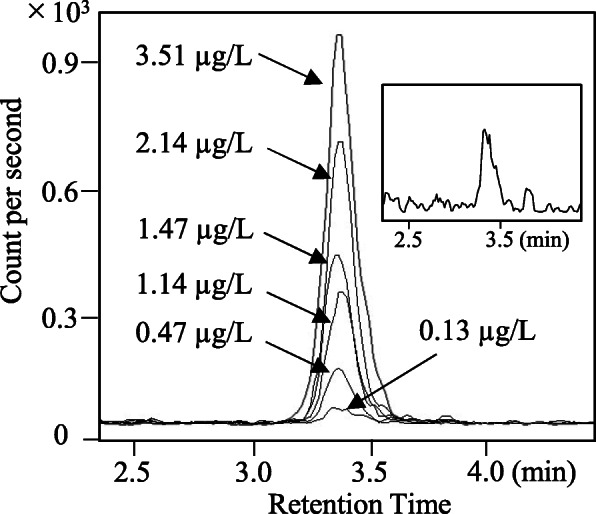
Mass chromatogram of the glyphosate at concentration range from 0.13 to 3.51 μg/L. The upper right chromatogram is the enlarged figure of the 0.13 μg/L chromatogram

The SPE procedure for glyphosate extraction was adopted in this study to ensure easy handling and high purification. As shown in Fig. 1, we used two types of solid-phase extraction columns. The good selectivity was suggested from the result of the mass chromatogram of pooled urine spiked with glyphosate at concentrations ranging from 0.13 to 3.51 μg/L (Fig. 3).

### Assay validation

Matrix effects data are shown in Table 3. The CreMDi_0.05_ method of all samples showed better %RSD values than the tenfold dilution.

**Table 3:** Absolute and relative matrix factors of 10 individuals

Sample ID	Creatinine (g/L)	Absolute matrix factors (row data of peak area)		Relative matrix factors (with IS collection)	
		Tenfold dilution	CreMDi_0.05_	Tenfold dilution	CreMDi_0.05_
1	1.28	0.57	0.69	1.10	0.90
2	0.48	1.02	0.75	1.02	0.92
3	0.32	0.95	0.79	1.18	1.07
4	1.13	0.60	0.83	0.95	1.03
5	0.45	1.76	0.90	1.51	1.10
6	1.62	0.51	0.72	1.34	0.92
7	0.79	0.84	0.77	1.97	1.10
8	0.21	1.12	0.83	1.03	1.21
9	0.69	0.49	0.72	1.57	1.02
10	1.37	0.82	0.52	1.34	0.80
%RSD		41.9	12.9	22.9	11.4

*CreMDi*_*0.05*_ creatinine-matching dilution to concentration at 0.05 g/L, *RSD* relative standard deviation

The absolute recovery rate, precision, and sensitivity parameters are summarized in Table 4. The absolute recovery rates ranged from 91.0 to 99.6%. For the within-run precision, the %RSD was 11.4% or less at a concentration of 0.33 μg/L. For the between-run precision, the %RSD was 14.0% at a concentration of 0.33 μg/L. The calibration curve with coefficients of determination (*r*^2^) was 0.998. The LOD and LLOQ values were 0.1 μg/L and 0.3 μg/L, respectively.

**Table 4:** Precision, recovery rate, linearity, LOD, and LLOQ data of the analytical procedure

	Concentration (μg/L urine)	*n*	Results
Absolute recovery rate			
(%)	0.33	3	99.6
	0.67	3	91.0
	1.33	3	97.8
Within-run precision			
(%RSD)	0.33	6	11.4
	0.67	6	8.6
	1.33	6	5.6
Between-run precision			
(%RSD)	0.33	5	14.0
	0.67	5	6.8
	1.33	5	9.6
Calibration curve			
Slope			0.144
Intercept			0.000
*r*^2^			0.998
LOD (μg/L, S/N = 3)			0.1
LLOQ (μg/L)			0.3

*n* number of observations, *RSD* relative standard deviation, *LOD* limit of detection, *LLOQ* lower limit of quantification, *S/N* signal-to-noise ratio

**Fig. 4:**
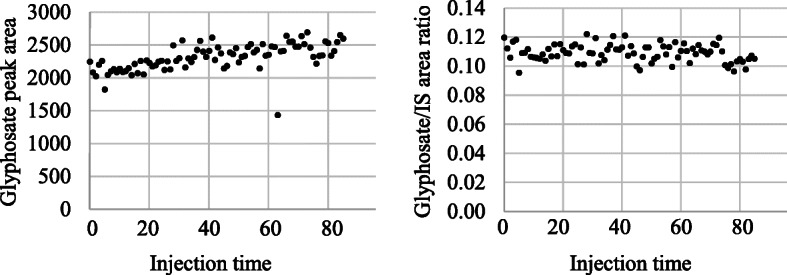
Variations of repeat injection from one vial containing prepared sample (0.67 μg/L of glyphosate). *X*-axis is the passage of time after the first analysis started, *Y*-axes are glyphosate peak area (left) or glyphosate/IS peak area ratio (right)

Figure 4 shows the result of eighty-six consecutive analyses within 22 h. Glyphosate peak areas were slightly varied and increased (left), while the glyphosate/IS peak area ratios were stable (%RSD was 5.6%) throughout the analysis (right). One possible cause of the increase in the peak area is the concentration of the prepared samples in the LC injector.

### Sample stability

The prepared samples were stable for at least 3 days at 0.67 μg/L concentration (Table 5). The storage stabilities of glyphosate in the urine were studied by storage for at most 1 week at 25 °C, or for at most 1 month at 4 °C or −80 °C, and three freeze-thaw cycles. Glyphosate in urine was stable through a few freeze-thaw cycles. Furthermore, the peak area ratios of glyphosate/IS in the stored urine was stable under each condition (Table 5).

**Table 5:** Glyphosate stabilities in urine

		Concentration (μg/L urine)	*n*	Storage period	% of 0 h
Prepared sample stability		0.67	2	0 h	
		0.67	2	18 h	96
		0.67	2	24 h	99
		0.67	2	48 h	98
		0.67	2	72 h	95
Freeze-thaw stability (three cycles)		0.67	3		92
Long-term stability	25 °C	0.67	3	1 week	94
	4 °C	0.67	3	1 week	92
		0.67	3	1 month	105
	−80 °C	0.67	3	1 week	105
		0.67	3	1 month	100

*n* number of observations

### Application of methods to urine samples

Our optimized method was applied to Japanese general people, and the resulting data are shown in Table 6. The detection frequencies (>LOD) of glyphosate were 70% for group A, 33% for group B, and 100% for group C. The maximum glyphosate values were 2.59 μg/g creatinine for group A, 0.68 μg/g creatinine for group B, and 1.72 μg/g creatinine for group C.

**Table 6:** Detection rates, geometric means, and percentiles of urinary glyphosate concentrations

Group	Sample size	>LOD (%)^a^	Units	GM	Selected percentiles					Max.
					5th	25th	50th	75th	95th	
A	20	70	μg/L	0.36	<LOD	<LOD	0.54	1.17	1.43	1.44
			μg/g cre	0.37	<LOD	<LOD	0.40	0.88	2.57	2.59
B	24	33	μg/L	NC^b^	<LOD	<LOD	<LOD	0.16	1.84	1.99
			μg/g cre	NC^b^	<LOD	<LOD	<LOD	0.34	0.65	0.68
C	10	100	μg/L	0.47	0.21	0.29	0.56	0.73	0.77	0.77
			μg/g cre	0.67	0.43	0.47	0.67	0.83	1.72	1.72
Total	54	59	μg/L	0.24	<LOD	<LOD	0.25	0.70	1.41	1.99
			μg/g cre	0.26	<LOD	<LOD	0.34	0.66	1.95	2.59

*LOD* limit of detection, *cre* creatinine, *GM* geometric mean, *NC* not calculated, *<LOD* lower than level of limit of detection

^a^Percent of detection frequency

^b^GM was not calculated due to low detection rate

## Discussion

There are two essential findings for the development of a quantitative method that is successfully applied to quantify low concentrations of urinary glyphosate with reliable analysis. First, low recovery and a reduction in the sensitivity of the phosphorylated compound glyphosate in LC-MS/MS analysis are mitigated by our novel approach using a metal-free analytical system with a metal deactivating agent and multi-mode columns. Second, the urine CreMDi_0.05_ method has enabled the stable and adequate reproducibility of glyphosate quantitative results not only from pooled urine samples but also from urine obtained from various persons.

Table 7 shows the comparative tables of determination method reports of urinary glyphosate using LC-MS/MS, including our present study. Each report has some strengths and weaknesses. For example, Jensen and colleagues reported the simplest sample preparation method [18]. Sample preparation of just dilution of urine may have a negative effect on LC-MS/MS sensitivity and reliability after repeated injection, leading to the anticipated system downtime to clean the MS ion source and HPLC system. Connolly and colleagues have successfully reported the urinary glyphosate concentration level in the general population using a commonly used LC-MS/MS system [14]. Unfortunately, the sensitivity and system validation assay data are insufficient. Our present method was optimized for sensitive and high-throughput application to many samples collected in an epidemiological study.

**Table 7:** Comparison of the present method with data obtained in two previously described methods

References	Jensen et al. 2016 [18]	Connolly et al. 2017 [14]	Present method
Analytical apparatus	LC-MS/MS (ESI)	LC-MS/MS (ESI)	LC-MS/MS (ESI)
Mass spectrometer	API5500 (ABSciex)	API3200 (ABSciex)	6430 (Agilent)
Analytical column	Cation-H 30 mm × 4.6 mm (Bio-Rad)	Zorbax SB-C3 150 × 4.6 mm 5 μm (Agilent)	Scherzo SM-C18 MF 100 × 2 mm 3 μm silica (Imtakt)
Sample preparation	Dilution	Solid-phase extraction (SAX)	Solid-phase extraction (SCX + NH2)
Internal standard	Glyphosate-^13^C_3_, ^15^N	Glyphosate-2-^13^C, ^15^N	Glyphosate-^13^C_2_, ^15^N
Amount of sample (μL)	600	500	ca. 100 (depend on the creatinine concentration)
Average recovery (%)	92-102 (0.1-800 μg/L, *n* = 6)		91.0-99.6 (0.33-1.33 μg/L, *n* = 3)
Intra-day precision (%RSD)	2.0-11.6 (0.1-800 μg/L, *n* = 6)	3.54 (*n* = 10)	5.6-11.4 (0.33-1.33 μg/L, *n* = 6)
Inter-day precision (%RSD)		9.96 (*n* = 40 over 4 runs)	6.8-14.0 (0.33-1.33 μg/L, *n* = 10 over 5 runs respectively)
LLOQ (μg/L)	0.10		0.3
LOD (μg/L)	0.023	0.5	0.1
Analytical stability			86 or more injection
Verification of matrix effect	Confirmed with pool urine		Confirmed with 10 individual urine
Prepared sample stability			72 h
Freeze-thaw sample stability	3 cycles^a^ (only human milk)		3 cycles
Long-term sample stability	5 °C (24 h)^a^ −20 °C (3 months)^a^		25 °C (1 week) 4 °C (1 month) −80 °C (1 month)
Application of methods to urine samples		*n* = 31, detection rate 45%, max 10.66 μg/L	*n* = 54, detection rate 59%, max 1.99 μg/L

^a^Matrix is human milk

It is well-known that the metal coordination compound glyphosate tends to cause tailing of peaks and intensity loss, resulting in low sensitivity and unreliable LC-based analysis. Moreover, the use of glass material has reduced the glyphosate in solution, due in part to ionic and/or hydrophobic adsorption to the siloxane and silanol. Therefore, metal- and glass-free analytical methods are essential for sensitive and reliable glyphosate analysis. As a countermeasure, an HPLC system with nonmetal tubing (mainly polyetherketone material) and/or capping the metal using phosphoric acid solution has been adopted in the glyphosate determination method [18]. Unfortunately, this countermeasure gave our laboratory far from satisfactory results in terms of reliability and sensitivity. As shown in Fig. 2, the mobile phase additive reagent MA Deactivator altered glyphosate sensitivity, leading to high reliability at low glyphosate concentration levels. This may be mainly due to the more inert environment for glyphosate by effective capping of free metal ions in the mobile phase and flow path, including the injection needle and analytical column, compared with phosphoric acid. Moreover, the Scherzo SM-C18 MF we selected provided a sufficient peak shape and high signal-to-noise ratio of glyphosate in chromatograms relative to previously reported column hydrophilic interaction chromatography (HILIC) [22] and an aqueous compatible reversed-phase [18] with whichever of some mobile phase (data not shown).

The CreMDi method provided satisfactory validation results caused by restraining the variation of matrix factors even in individual urine samples. The creatinine concentration reflects the degree of urine concentration, which has intra- and inter-individual differences ranging from 0.21 to 5.44 g/L in the study subjects. It is necessary to standardize the amount of substances in urine to ensure the equal effect of glyphosate purification by the SPE column. A complicated liquid handling is needed for the CreMDi_0.05_ method. However, the CreMDi method also has the advantage of assigning approximately the same LOD or LLOQ values to individual urine samples, with the same background chromatogram and unifying matrix effects.

Although the procedure was complicated for the two types of SPE, the time required for sample processing was short because the test design allowed the SPE operation using an automatic device (Extrahera™). A combination of strong cation exchange and a weak anion-exchange column (SCX and NH2) was selected as a better preanalytical procedure for our LC-MS/MS condition. The preanalytical procedure using two types of SPE columns involves a cost relative to the other method [14, 19]. Addressing this disadvantage was expected to bring benefits in terms of the chromatogram peak specificity (Fig. 3) and stable sensitivity evidenced by the repetition test of sample injection (more than 86 injection, Fig. 4). In this study, glyphosate metabolite aminomethylphosphonic acid was excluded from the urinary target biomarker because of its low urinary excretion rate [7]. However, there are reports that glyphosate and aminomethylphosphonic acid have a correlation with urinary excretion concentration [10]. Therefore, we will consider glyphosate and aminomethylphosphonic acid as analytes in the future.

The present method has been successfully applied to urine samples of Japanese children, adults, and farmers. This implies that our method can be applied to the HBM of urinary glyphosate even at environmental exposure levels. Statistical comparison among the three groups was not performed due to the small sample size. Table 8 shows the median and maximum levels of urinary glyphosate in the general population reported within 10 years. The urinary glyphosate concentration of the Japanese seems to be approximately the same as or lower than that of German or Irish studies. However, the results of the urinary concentration of glyphosate in our study are not conclusive as representative Japanese data because of the small sample size and limited sampling area. Above all, the results of this study strongly suggest that our method can be readily applied to biomonitoring of urinary glyphosate in general populations. To assess the precise exposure of glyphosate using human biomonitoring, a further study is needed to collect basic information, including the relationship between intake and excretion of glyphosate.

**Table 8:** Urinary glyphosate concentrations reported in previous papers

Author, sampling country, sampling year	Study population (years)	Sample size	LOD (μg/L)	Detection rate	Units	Median	Max.
Knudsen et al., Denmark, 2011 [11]	Children (6-11)	14	NR	100%	μg/L	1.96	3.31
	Mothers (31-52)	13	NR	100%	μg/L	1.28	3.22
Conrad et al., Germany, 2015 [10]	Adults (20-29)	399	0.1 (LLOQ)	31.8%	μg/L	NR	2.80
Jayasmana et al., Sri Lanka, NR [12]	Adults (39.3 ± 11.5)	10	0.6	NR	μg/L	3.3	5.5
					μg/g cre	2.4	4.4
Parvez et al., USA (Indiana), 2015-2016 [13]	Pregnant Women (18-40)	71	0.1	93%	μg/L	3.25^a^	7.20
Connolly et al., Ireland, 2017 [14]	Farmer workers (33-66)	31	0.5	45%	μg/L	1.35^b^	10.66

*LOD* limit of detection, *LLOQ* lower limit of quantification, *NC* not calculated, *NR* not reported, *cre* creatinine

^a^Median concentration except for <LOD samples

^b^Arithmetic mean

## Conclusion

We have developed a highly sensitive and reliable method for the quantitation of urinary glyphosate, and we have applied this method to detect glyphosate in the urine samples of young Japanese children and adults. The present study is the first to report urinary concentrations and glyphosate distributions in the Japanese people. Although much information about the glyphosate exposure level remains poorly understood, this promising method would contribute to the development of future research on risk assessment from glyphosate exposure in the general population, including children and adults with occupational exposure.

## Data Availability

Due to the nature of this research, participants of this study did not agree for their individual data to be shared publicly, so supporting data is not available.

## Abbreviations

CreMDi_0.05_: Creatinine-matching dilution to 0.05 g/L
ELISA: Enzyme-linked immunosorbent assay
ESI: Electrospray ionization
FCMIA: Fluorescence covalent microbead immunosorbent assay
GC: Gas chromatography
GM: Geometric means
HBM: Human biomonitoring
HILIC: Hydrophilic interaction chromatography
HPLC: High-performance liquid chromatography
IS: Internal standard
LC: Liquid chromatography
LLOQ: Lower limit of quantification
LOD: Limit of detection
MA: Medronic acid
MRM: Multiple reaction monitoring
MS: Mass spectrometry
NCI: Negative chemical ionization
RSD: Relative standard deviation
SPE: Solid-phase extraction
S/N: Signal-to-noise ratio
